# Guideline Adherence of Asymptomatic Bacteriuria Management Among Physicians in Jordan: A Cross-sectional Study

**DOI:** 10.1093/ofid/ofaf254

**Published:** 2025-06-16

**Authors:** Hadeel Allan, Thekraiat Al Quran, Othman Beni Yonis, Wasan Alzu'bi

**Affiliations:** Public Health Department, Faculty of Medicine, Jordan University of Science and Technology, Irbid, Jordan; Public Health Department, Faculty of Medicine, Jordan University of Science and Technology, Irbid, Jordan; Public Health Department, Faculty of Medicine, Jordan University of Science and Technology, Irbid, Jordan; Public Health Department, Faculty of Medicine, Jordan University of Science and Technology, Irbid, Jordan

**Keywords:** antimicrobial resistance, asymptomatic bacteriuria, clinical practice, guideline adherence, urinary tract infection

## Abstract

**Background:**

Asymptomatic bacteriuria (ASB) is the presence of bacteria in urine without symptoms of a urinary tract infection. The management of ASB varies widely among health care providers, particularly in different regional contexts. This study aims to assess guideline adherence for ASB management among physicians in Jordan.

**Methods:**

A cross-sectional study was conducted among physicians from various medical specialties in Jordan between January and March 2024. A total of 750 surveys were distributed to participants through email and phone channels, focusing on demographic information, clinical practices, and adherence to available ASB management guidelines. Of these, 418 responses were received, yielding a response rate of 55.7%. The collected data were subsequently compiled and analyzed.

**Results:**

In total, 418 survey responses were analyzed. Participants included general practitioners, urologists, obstetricians/gynecologists, surgeons, internal medicine specialists, family medicine practitioners, and residents. There were significant deviations from recommended guidelines, particularly in antibiotic selection and treatment duration. Ciprofloxacin was the most preferred antibiotic (34.2%), contrary to guidelines recommending nitrofurantoin or trimethoprim-sulfamethoxazole.

**Conclusions:**

The findings highlight the need for improved adherence to ASB management guidelines among Jordanian physicians. Educational interventions and policy implementations are essential to optimize clinical care and reduce antimicrobial resistance.

Asymptomatic bacteriuria (ASB) is defined as the presence of bacteria in the urine without symptoms of a urinary tract infection (UTI) [1].

UTIs typically present with symptoms such as dysuria (painful urination), increased frequency of urination, urgency, suprapubic pain, and hematuria (blood in urine) [2]. These symptoms are absent in ASB, making it crucial to differentiate between symptomatic bacteriuria and ASB to avoid unnecessary antibiotic use [1].

Although ASB is usually not clinically apparent, it poses significant management challenges in health care, especially considering its prevalence among specific groups, such as the elderly, pregnant women, and patients with chronic urologic conditions [3]. Globally, prevalence rates range from 1% to 15%, varying by patient demographics and clinical settings [4]. The condition is recognized as a risk factor for developing symptomatic UTI, pyelonephritis, and adverse pregnancy outcomes, including preterm birth and low birth weight [4, 5].

Guidelines for ASB management, such as those from the Infectious Diseases Society of America (IDSA), generally advise against routine screening and treatment of ASB in most populations, except for high-risk groups such as pregnant women and individuals undergoing urologic procedures [3, 6, 7]. Treating ASB without clear indications does not improve clinical outcomes and may contribute to increased antimicrobial resistance [3]. Despite these guidelines, there are observed discrepancies in clinical practices, especially in regions with varying health care structures and resources [8].

In Jordan, limited studies have been conducted to assess the adherence of health care providers to ASB management guidelines. This study aims to evaluate the practices of physicians in Jordan regarding ASB management to identify adherence levels to international guidelines. Understanding these factors is critical for enhancing patient care and mitigating the risks associated with inappropriate antibiotic use.

## METHODOLOGY

This study employed a cross-sectional survey design to evaluate physician adherence in Jordan to the 2019 IDSA guidelines for the management of ASB. Data were collected via an online survey distributed to 750 physicians across various medical specialties, including general practitioners, urologists, obstetricians/gynecologists, surgeons, internal medicine specialists, family medicine practitioners, and residents. A total of 418 responses were received, yielding a response rate of 55.7%.

The survey included questions on demographic information, clinical practices related to ASB management, and adherence to specific guidelines. Participants were recruited through professional networks, medical associations, and online platforms, ensuring a diverse representation of physicians across specialties and practice settings (Table 1).

**Table 1.** Findings on Guideline Adherence for Asymptomatic Bacteriuria Management Among Physicians in Jordan

Section	Finding	Implication
Demographics	Total participants: 418 Family medicine: 25.6% General practitioners: 18.7% Internal medicine: 14.5% Surgeons: 13.2% Obstetricians/gynecologists: 11.3% Urologists: 8.7% Residents: 8%	Diverse representation across specialties; most physicians work in health care centers (57.9%) and hospitals (30.3%).
Screening practices	36.8% of participants do not routinely screen for asymptomatic bacteriuria. Others screen for specific patient groups: pregnant women (63.2%), patients with indwelling urinary catheters (19.7%), those scheduled for urologic procedures (30.3%).	Variation in screening practices based on patient risk factors; potential for improvement in broadening screening criteria.
Diagnostic methods	Urinalysis + culture: 55.3% Urinalysis only: 26.3% Culture only: 18.4%	Indicates a reliance on varying diagnostic standards; potential for improvement in diagnostic accuracy.
Follow-up practices	35.5% do not routinely follow-up; 23.7% use urinalysis + culture; 18.4% use urinalysis only.	Lack of consistent follow-up practices may lead to unresolved cases and undetected resistance issues.
Antibiotic preferences	Ciprofloxacin: 34.2% Nitrofurantoin: 31.6% Trimethoprim-sulfamethoxazole: 25%	Preference for ciprofloxacin diverges from guidelines recommending nitrofurantoin or trimethoprim-sulfamethoxazole.
Treatment duration	1–3 d: 9.2% 4–7 d: 81.6% 8–14 d: 7.9%	Majority follow a common treatment duration; however, appropriateness varies by individual cases.
Utilization of guidelines	34.2% follow no specific guidelines; 35.5% adhere to CDC guidelines; 18.4% follow local protocols; 2% comply with IDSA guidelines; 8.9% use other resources.	Significant nonadherence highlights a critical gap in practice that could be addressed through education and policy changes.
Clinical scenario response	61.8% would treat a 75-y-old woman with significant bacteriuria but no urinary tract infection symptoms.	Indicates a concerning trend of initiating unnecessary antibiotic treatment contrary to guidelines.
Perception of resistance	43.4% encounter antibiotic resistance occasionally.	Awareness of resistance issues is present but not sufficiently integrated into prescribing practices.
International comparison	Similar nonadherence trends noted globally (eg, US, 45%; Canada, 32%).	Reflects a widespread challenge in adhering to asymptomatic bacteriuria management guidelines that may require systemic changes.
Recommendations	Implement educational interventions and policy updates to promote guideline adherence.	Essential for optimizing patient care and addressing the threat of antimicrobial resistance.

Abbreviations: CDC, Centers for Disease Control and Prevention; IDSA, Infectious Diseases Society of America.

Data analysis was performed with descriptive statistics to summarize the survey responses. Ethical approval was obtained from the Jordanian institutional review board, and participation was voluntary, with confidentiality maintained throughout the study.

## RESULTS

The study findings and their implications in ASB management practice are summarized in Table 1.

### Demographic Characteristics

**Figure 1.**
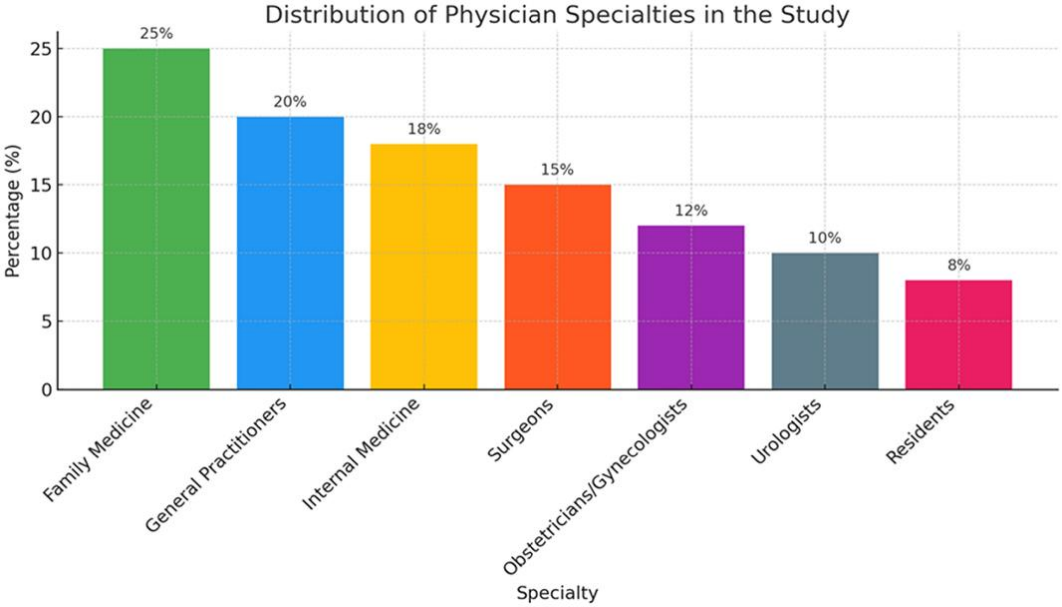
The distribution of physician specialties and their experience range from the study.

A total of 418 physicians participated in the study. Family medicine practitioners composed the largest group, accounting for 25.6% of the participants, followed by general practitioners (18.7%), internal medicine specialists (14.5%), surgeons (13.2%), obstetricians/gynecologists (11.3%), urologists (8.7%), and residents (8%). Participants’ experience varied from 3 to 20 years (Figure 1).

Most participants worked in health care centers (57.9%), followed by hospitals (30.3%), private clinics, and other settings. The diversity of practice environments provided a comprehensive overview of ASB management practices across Jordan's health care system.

### Clinical Practices

The survey revealed variability in the diagnostic methods used for ASB.

Overall, 36.8% of participants do not routinely screen for ASB; others reported screening for specific patient groups, including pregnant women (63.2%), patients with indwelling urinary catheters (19.7%), and those scheduled for urologic procedures (30.3%). Factors influencing the decision to initiate antibiotic treatment included symptoms reported by the patient (63.2%), the presence of underlying conditions (eg, diabetes or immunocompromised status; 56.6%), and pregnancy (81.6%).

The majority of physicians (55.3%) preferred a combination of urinalysis and urine culture to detect ASB, while 26.3% relied solely on urinalysis and 18.4% urine culture as their primary diagnostic tool. Interestingly, 81.3% of participants did not consider repeating the test after a single positive result for ASB, indicating a potential area for practice improvement.

When it came to antibiotic preferences, ciprofloxacin was the most commonly chosen antibiotic (34.2%), followed by nitrofurantoin (31.6%) and trimethoprim-sulfamethoxazole (25%). These findings highlight a deviation from the IDSA guidelines, which emphasize that the choice of first-line agents for ASB management depends on the clinical scenario. For instance, in pregnant patients with ASB, nitrofurantoin and β-lactam antibiotics, such as ampicillin or cephalexin, are typically recommended [3].

The duration of antibiotic treatment varied, with the majority (81.6%) prescribing antibiotics for 4 to 7 days. A smaller proportion prescribed antibiotics for 1 to 3 days (9.2%) or 8 to 14 days (7.9%). Follow-up practices after treatment completion were inconsistent: 35.5% of physicians did not routinely conduct follow-up, while others used methods such as urinalysis combined with culture (23.7%) or urinalysis alone (18.4%). According to established guidelines, such as those from the IDSA, ASB should not be treated or followed with repeat urine cultures except in specific populations (eg, pregnant individuals or those undergoing urologic procedures). Therefore, the lack of repeat testing in most cases is considered guideline adherent.

### Utilization of Guidelines

**Figure 2.**
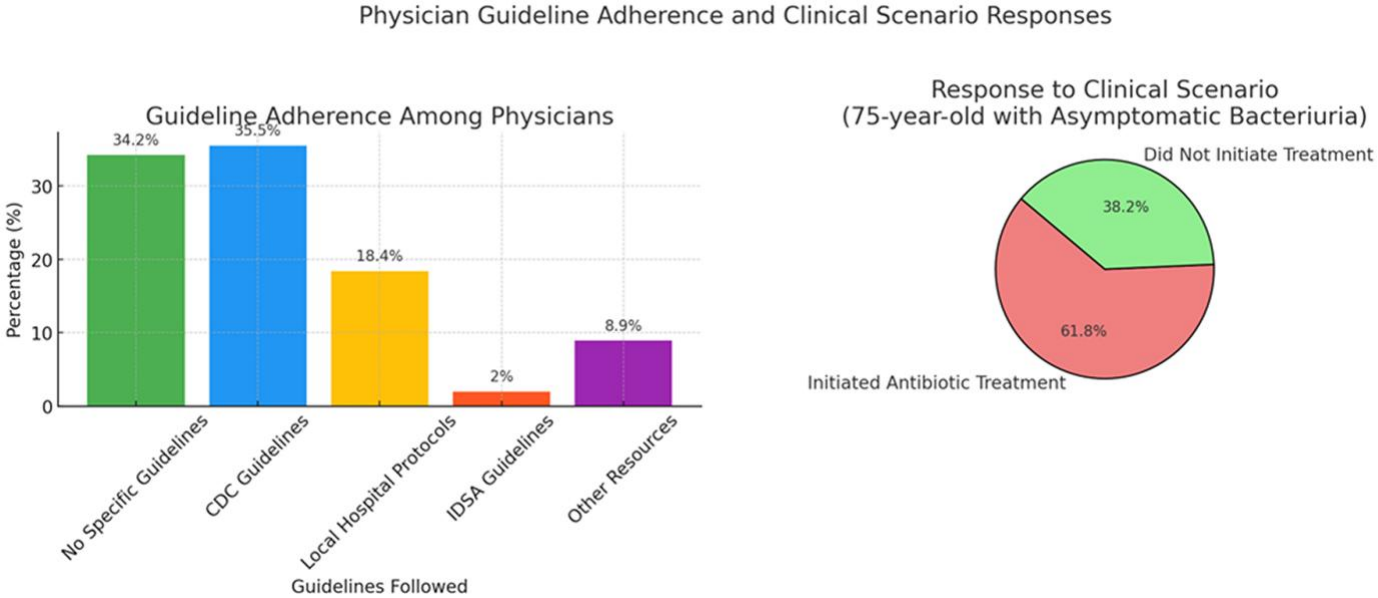
Physician guideline adherence in the management of asymptomatic bacteriuria. Abbreviations: CDC, Centers for Disease Control and Prevention; IDSA, Infectious Diseases Society of America.

Guideline adherence among physicians varied significantly (Figure 2). About 34.2% reported not following any specific guidelines when managing ASB; 35.5% adhered to guidelines of the Centers for Disease Control and Prevention; 18.4% followed local hospital protocols; 2% complied with IDSA guidelines; and 8.9% used other resources such as UpToDate. In response to a clinical scenario involving a 75-year-old woman with significant bacteriuria but no symptoms of a UTI, 61.8% of participants opted to initiate antibiotic treatment (Figure 2), demonstrating a divergence from recommended practices [3, 6].

## DISCUSSION

The study reveals significant variability in the management of ASB among Jordanian physicians, demonstrating a prevalent use of antibiotics that are not recommended by current guidelines and have inconsistent treatment durations. A noticeable preference for ciprofloxacin, despite clinical guidelines recommending nitrofurantoin or trimethoprim-sulfamethoxazole as first-line agents, suggests that antibiotic selection practices may be influenced by local prescribing habits, patient expectations, and limited access to updated clinical guidelines. These findings reflect broader, systemic issues that align with international data, where deviations from recommended practices are attributed to factors such as health care provider education, cultural influences, and availability of resources [9–11].

Comparative international analyses indicate similar challenges in adherence to ASB management guidelines. In the United States, studies have shown that only 45% of physicians strictly follow the IDSA recommendations, with ciprofloxacin frequently overprescribed despite not being recommended as a first-line treatment [9]. A systematic review and meta-analysis of studies across multiple countries revealed that inappropriate management of ASB is widespread, with overtreatment rates. The review found that antibiotics were frequently prescribed even in the absence of symptoms, particularly in inpatient and long-term care settings, indicating a significant gap between evidence-based guidelines and clinical practice [12]. In Canada, adherence rates among general practitioners are even lower—approximately 32%—with significant regional variations driven by local practices and access to updated guidelines [10]. Similarly, European studies demonstrate inconsistent adherence, as influenced by cultural norms, health care system differences, and variable dissemination of guideline updates [11]. Altogether, the high prevalence of inappropriate antibiotic use in ASB management underscores a critical need for targeted educational interventions and policy reforms to enhance adherence to evidence-based guidelines and mitigate the growing threat of antimicrobial resistance.

This study has notable strengths. First, inclusion of participants from across regions of Jordan allowed for a more representative understanding of national clinical practices. Additionally, the survey captured responses from a variety of provider types—including general practitioners, specialists, and trainees—enhancing the generalizability of the findings. However, the study is subject to limitations, such as potential response bias due to self-reporting. Future studies incorporating interviews or focus groups may provide deeper insights into the motivations behind clinical decision making.

## CONCLUSION

This survey highlights the need for improved adherence to ASB management guidelines among physicians in Jordan. The observed deviations from recommended practices, particularly antibiotic selection and treatment duration, suggest a critical need for educational initiatives, workshops, and updated clinical protocols to enhance the management of ASB. Implementing these changes will be crucial in optimizing patient care and combating the growing threat of antimicrobial resistance.

## Notes

This article has been read and approved by all the authors, and each author believes that the article represents honest work.

***Data-sharing statement.*** Deidentified data are available upon reasonable request to Dr Hadeel Allan (hadeel_allan25@yahoo.com) after execution of a data use agreement with the Jordan University of Science and Technology.

***Patient consent information.*** Our study does not include factors necessitating patient consent. The design of the work has been approved by the institutional review board at the Jordan University of Science and Technology.
